# Exploring the potential of low doses carbon monoxide as therapy in pregnancy complications

**DOI:** 10.1186/2045-9912-2-4

**Published:** 2012-02-20

**Authors:** Tarek El-Mousleh, Pablo A Casalis, Ivonne Wollenberg, Maria L Zenclussen, Hans D Volk, Stefanie Langwisch, Federico Jensen, Ana C Zenclussen

**Affiliations:** 1 Department of Experimental Obstetrics and Gynecology, Medical Faculty, Otto-von-Guericke University, Magdeburg, Germany; 2 Institute of Medical Immunology, Charité, Universitätsmedizin, Berlin, Germany; 3 Berlin-Brandenburg Center for Regenerative Therapies, Charité, Universitätsmedizin, Berlin, Germany

## Abstract

Heme Oxygenase-1 (HO-1) has been shown to play a pivotal role in pregnancy outcome and its ablation leads to abnormal placentation, intrauterine fetal growth restriction (IUGR) and subsequent intrauterine fetal death. Carbon monoxide (CO) has been found to mimic the protective effects of HO-1 activity, rescuing HO-1-deficient fetuses. This gasotransmitter arises in biological systems during the oxidative catabolism of heme by HO. Here, we explored the potential of CO in preventing IUGR and established the optimal doses and therapeutic time window in a clinically relevant mouse model. We additionally investigated the pathways activated upon CO application *in vivo*. We established 50 *ppm *as the best lowest dose of CO necessary to prevent growth restriction being the optimal time frame during days 3 to 8 of mouse pregnancy. CO lead to higher fetal and placental weights and avoided fetal death without showing any pathologic effects. CO breathing further suppressed inflammatory responses, diminished placenta apoptosis and complement deposition and regulated placental angiogenesis. Our results confirm the protective role of the HO-1/CO axis and point this gas as an emerging therapeutic possibility which is worth to further explore.

## Introduction

In mammals, most embryonic losses occur during early pregnancy [1,2], which represents a critical period of gestation because of the major developmental events that take place, including placentation and embryonic organogenesis [3-5]. Placentation comprises extensive angiogenesis in maternal and placental tissues, accompanied by a marked increase in uterine and umbilical blood flows [6,7]. Reduced placental vascular development and increased vascular resistance are associated with early embryonic mortality [8,9]. Factors influencing placental vascular development and function have a dramatic impact on fetal growth and development, and thereby on neonatal survival and growth [10,11]. We have recently identified the enzyme heme oxygenase-1 (HO-1) as a pivotal factor in supporting placentation [12]. The protective effects of HO-1 on placentation can be mimicked by administering mice partially deficient in HO-1 (*Hmox1*^+/-^) with carbon monoxide (CO, 12). Exogenous supply of CO at low concentrations can regulate many physiological processes without apparent toxicity and is able indeed to restore the immunoregulatory and cytoprotective effects of HO-1 after its pharmacologically inhibition in a variety of pathologies [13-15]. This gasotransmitter is an endogenous product of heme degradation through HO-1. CO has been shown to exert cytoprotective effects by reducing pro-inflammatory mediators, preventing vascular constriction, decreasing platelet aggregation and inhibiting apoptosis [13] and was proposed to be a placenta vasodilator [16]. CO has been also implicated in the angiogenic response associated with induction of HO-1 [14,15].

Having learned that HO-1 is crucial for placentation and intrauterine fetal survival via CO, we aim here to investigate the therapeutic potential of CO in avoiding pregnancy complications. To do so we established the optimal doses and time frame of treatment with CO via inhalation in a clinically relevant model of intrauterine growth restriction (IUGR). We analyzed the possible toxic effects of the therapy as well as the pathways activated at throphoblast level after CO application.

## Materials and methods

### Mice

CBA/J, DBA/2J and BALB/c mice were obtained from Charles River, Sulzfeld, Germany, and maintained in our animal facilities in Berlin and Magdeburg, Germany with a 12 h light/dark cycle with water and food *ad libitum*. Experimental procedures were approved by the German authorities (LaGeSo Berlin 0062/03 and Landesverwaltungsamt Sachsen-Anhalt AZ: 2-868 University of Magdeburg). We performed our experiments using the well-established combination CBA/J x DBA/2J, which has been recently described as a suitable IUGR model [17]. Mating of CBA/J females with BALB/c males served as a control combination as it represents a normal pregnancy. Two months old CBA/J females were mated with 2-4 months-old BALB/c or DBA/2J males, checked twice a day for vaginal plugs and separated from the males if pregnant. The day of the vaginal plug was considered as day 0 of pregnancy. Animals were treated with mixed air (20.9% O_2_, pharmaceutical compressed air) or CO as indicated below.

### CO exposure

Mice were placed in a 98-liter Plexiglas animal chamber (A-Chamber, BioSpherix, NY, USA) and exposed to CO (50 or 125 parts per million, *ppm *mixed with air) during either days 3 to 5 or 3 to 8 of pregnancy as explained elsewhere [12]. Control mice were maintained in a similar chamber without CO, only receiving the mixed air. The gas flow into the Plexiglas chambers was maintained continuously at a rate of 12 liter/min. CO at a concentration of 5% (50.000 *ppm*) in balanced air (20.9% oxygen) was mixed with compressed air to obtain a final concentration of 50 or 125 *ppm *before being delivered into the exposure chamber. The compressed air came from a 7 bar in house air-supply system and the CO from a high-pressure bottle (Linde Gas Therapeutics GmbH, Unterschleißheim, Germany). CO concentration was controlled by varying the flow rate of CO using a flowmeter (Q-Flow, Vögtlin Instruments, Switzerland) before delivering to the chamber. Because the flow rate (12 liter/min) is primarily determined by the air flow, only the CO flow was changed to deliver the final concentration to the exposure chamber. A CO monitor (X-am 2000 Multi-gas Monitor, Dräger, Germany) was used to measure CO levels in the chamber. Gas samples were introduced to the monitor through a port in the side of the chamber and were analyzed by electrochemical detection (DrägerSensor XXS CO - 68 10 882, Dräger, Germany). Concentration levels were measured periodically and there were no fluctuations in the CO concentration after the chamber had equilibrated (approximately 10 min). The chambers were housed in a fume hood during the experiments and the room was equipped with a CO alarm. In the chambers, gas flowed at controlled rates to obtain the desired CO exposure conditions.

### Experimental settings: Animal groups

For analysing the effect of CO on pregnancy outcome in mice known to develop IUGR, CBA/J females mated with DBA/2J males (n = 8/ group) were treated with different CO doses (50 and 125 *ppm*) as follows:

a) 125 or 50 *ppm *of CO were applied during implantation window (days 3-5 *postcoitum*, dpc)

b) 50 *ppm *of CO were applied during implantation and early placenta development (3-8 dpc)

CBA/J animals previously mated with BALB/c males (normal pregnancy) were treated with 50 *ppm *CO to study the effect of this gas in normal pregnancies. This was mainly due to discard toxic effects of CO in the normal pregnancy combination.

Implantation rate as well as the percentage of fetal death was analyzed at day 14 of pregnancy after sacrificing the animals by calculating the percentage of non-viable implantations to the total number of implantations (viable + non-viable) multiplied by 100. Placentas and fetuses were weighted. Tissue samples were obtained for flow cytometry, real-time-PCR, histopathology and immunohistochemistry studies.

In a second experiment, females of the IUGR combination (n = 8) were treated with 50 *ppm *of CO during days 3-8 being the animals sacrificed directly after CO exposure (day 8) to analyze the immediate effect of CO application at trophoblast level.

### Measurement of Carboxyhemoglobin (COHb)

Blood was taken from the heart of the anesthetized animal and COHb as well as total hemoglobin (tHb) were analyzed by a blood gas analyzer (ABL 520, Radiometer, Copenhagen).

### Flow cytometry

Cells isolated from spleen, lymph nodes or decidua (18) were incubated for 1 hour with 50 ng/ml phorbol 12-myristate 13-acetate (PMA) and 1 μg/ml ionomycin at 37°C with 5% CO_2 _for stimulation of cytokine secretion. To allow intracellular accumulation of secreted proteins 2 μM of monensin was added and incubated for further 3 hours. Cells were washed and incubated with antibodies against surface markers for 10 min at 4°C in darkness. For fixation, paraformaldehyde solution (PFA) at a concentration of 1% (p/v) was used, and cells were incubated overnight (ON) at 4°C. For detecting intracellular proteins, cells were washed and antibodies diluted in saponin 0.1% (p/v) were incubated for 20 min at 4°C in darkness. After the incubation time, cells were washed with saponin solution. The labelled cells were finally resuspended in FACS buffer and analyzed in a FACS Calibur (Becton Dickinson) cytometer. The lymphocyte population was gated based on size and granularity and used for further analysis. When only analysing extracellular markers, incubation with PMA, ionomycin and monensin was avoided. Antibodies used included: FITC-labelled anti-CD4; PE-labelled anti CD25, TNF-α, IFN-γ and IL-17; PE-Cy7-labelled anti CD3 (all purchased from BD Biosciences, Heidelberg, Germany).

### Immunohistochemistry (IHC)

Placenta samples were fixed in 96% ethanol and embedded in paraffin as described by Saint-Marie (see [19]). For detecting VEGF, paraffin samples were dewaxed with xylol (2 × 20 min) followed by a series of incubations in ethanol (100%, 95% and 75%, 10 min each), with a final incubation of 5 min in distilled water. The sections were washed with Tris buffered saline solution (TBS, pH = 7.40) for 10 min and treated with 3% hydrogen peroxide in methanol for 30 min at room temperature (RT) to block the endogenous peroxidase activity. After washing, the tissues were exposed to 5% BSA in TBS for 20 min at RT for protein blocking, stained with the primary antibody (goat anti-VEGF, Santa Cruz, San Diego, USA) diluted 1:100 in 5% BSA in TBS and incubated ON at 4°C. The tissues were washed and further stained with the secondary antibody (rabbit anti-goat, Dako, Germany) diluted in 5% BSA in TBS for 1 h at RT. After washing, the samples were incubated for 30 min with an AB-Complex/HRP solution (Dako, Germany). Finally, they were developed with AEC+ Substrate Chromogen (Dako), counterstained with Hematoxylin and mounted. Negative controls were obtained by replacing the first antibody with 5% BSA or goat diluted serum. For complement staining, 8 μm thick sections were cut, fixed and incubated with goat anti-mouse C3 (Cappel) 1:200, diluted in 5% BSA in TBS for 60 min at RT. As secondary antibody, we employed rabbit anti-goat biotinylated 1:200 for 60 min at RT. After washing, the samples were incubated for 30 min with an AB-Complex/HRP solution, developed with AEC+ Substrate Chromogen, counterstained with Hematoxylin and mounted.

### TUNEL assay

Paraffin-embedded placenta sections were dewaxed and hydrated as already described. This was followed by a permeabilization of the samples with 0.1 M citrate buffer. The detection of apoptotic cells was performed following manufacturers instructions using the *in situ *Cell Death Detection Kit, POD (Roche Diagnostic Systems, Mannheim, Germany) but changing the incubation temperature to RT instead of 37°C. Amino-9-ethylcarbazole/substrate chromogen (DakoCytomation, Eching, Germany) and a counterstaining with Hemalaun (Roth, Karlsruhe, Germany) was used for visualization of apoptotic cells. Negative controls were performed by using only the label solution. All sections were analyzed under the light microscope by two independent observers without knowledge of the samples. The numbers of TUNEL^+ ^cells per mm^2 ^of placental tissue were evaluated with a scaled eyepiece, pre-calibrated with a slide micrometer.

### Protein isolation and western blot analysis

Frozen placenta pieces were homogenized in lysis buffer (1% NP-40, 0,1 mg/ml n-Dodecil beta maltoside, 10 mM NAO3V, 1 M Tris pH 7.5, 5 m NaCl, 500 mM NaF, 500 mM EDTA pH 7,5, 100 mM PMSF) for 45 min. After isolation, homogenates were centrifuged at 12.000 rpm for 20 min at 4°C and the supernatant containing the proteins was transferred to a fresh tube. Protein concentration was assessed using the Pierce BCA Protein Assay (Thermo Thermo Fisher Scientific, Bonn, Germany) as indicated by the manufacturer. Protein samples were kept at -80°C and while working with them, they were always kept on ice. For Western Blot analysis, 20 μg of protein (for Bag-1, sFlt-1 and sEng analysis) or 50 μg of protein (for VEGF analysis) were transferred into a 8% (sFlt-1), 10% (Bag-1 or sEng) or 15% (VEGF) polyacrylamide gel and a SDS-PAGE in denaturizing conditions was performed at 100 V. After the electrophoresis proteins were transferred into PVDF membranes in transfer buffer containing 20% methanol (v/v), 0,19 M glycine and 0.025 M Tris-base pH 8,3. For protein detection, membranes were incubated with primary antibodies for 2 h with rabbit polyclonal antibody against Bag-1 (1:500), or overnight (ON) with goat polyclonal antibody against VEGF (1:100), sFlt-1 (1:200) or sEng (1:200) all from Santa Cruz, Biotechnology, San Diego, USA. After three washing steps with TBST (TBS with 0.5% Tween) for 5 min each, the membranes were then incubated with an anti rabbit HRP-conjugated (Thermo Fisher Scientific) or anti goat biotin-conjugated (Dako) antibody diluted 1:2000 for 1 h at RT and then with avidin-horseradish peroxidase complex (ABC complex, Biozol). GAPDH or β-Actin was used as loading control. The chemiluminescence signal was generated by using luminol (A8511-5G, Sigma-Aldrich), 4-hydroxycinnamic acid (p-coumaric acid; C9008-25G, Sigma Aldrich), and hydrogen peroxide (Merck, Darmstadt, Germany). The intensity of the bands was quantified by using the GeneSnap^® ^Software, Version 4.01c from Syngene.

### RNA isolation, cDNA synthesis and Real-time RT- PCR

RNA extraction was performed using Trizol^® ^Reagent (Invitrogen, Darmstadt, Germany). Briefly, frozen placentas (100 mg) were treated with 1 ml Trizol^® ^and disaggregated using an Ultra-Turrax T25 homogenizer. Isolation of RNA, cDNA synthesis and RT-PCR was performed as described elsewhere [18]. For Bag-1 Bcl, Bclxl, Bax, RT-PCR was performed using TaqMan technology. For amplifying VEGF, PGF, SDF-1α and HIF-1α, RT-PCR was performed using SYBR green technology (Applied Biosystems, Warrington, UK), both with the i-Cyler (Biorad, Munich, Germany). β-Actin was used as house keeping gene. The amount of mRNA was calculated as 2^-ΔCt ^in both cases. Primer sequences are available upon request.

## Results

### 50 ppm CO application during days 3-8 of pregnancy was effective in preventing fetal loss while not provoking deleterious effects in mothers or pups

**Figure 1:**
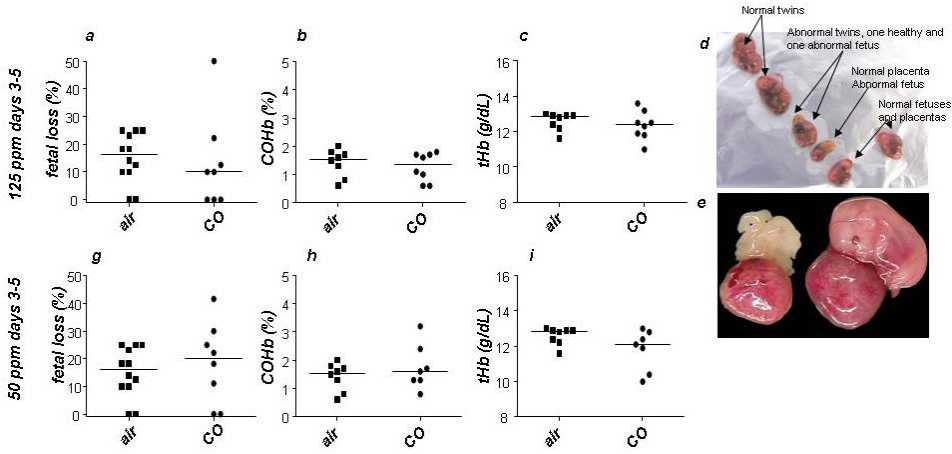
**Effect of moderate (125 *ppm*) and low (50 *ppm*) CO concentration during implantation window on murine pregnancy outcome**. (a) Pregnant mice treated with 125 ppm of CO during implantation window (days 3-5 of pregnancy) show sligthly diminished fetal loss rate compared to air-treated controls as analyzed on day 14 of pregnancy (b) Maternal COHb and (c) tHb levels were comparable among the groups. (d, e) Treatment with 125 *ppm *of CO provoked the pregnant mice to carry abnormal fetuses (d, e). Exposure of pregnant females to 50 *ppm *of CO during days 3-5 of pregnancy did not modify pregnancy outcome when compared to air-treated controls (g). Neither COHb (h) nor tHb levels (i) in maternal blood were modified by this treatment. The data are shown as medians. *p < 0.05, as analyzed by the non-parametric Mann-Whitney *U*-test between two groups.

**Figure 2:**
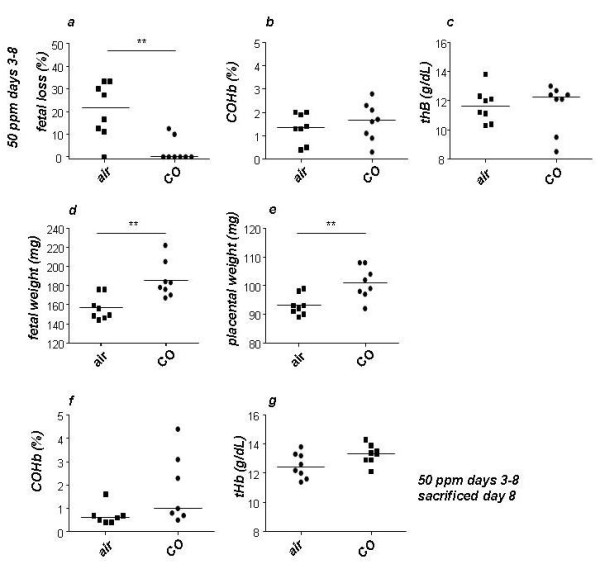
**CO treatment applied during embryo implantation and early placentation positively influence pregnancy outcome**. (a) Inhalation of low amounts of CO (50 *ppm*) during implantation and early placentation (days 3-8) completely prevented fetal loss as analyzed on day 14 of pregnancy. COHb (b) and tHb (c) levels in maternal blood were not modified by the treatment. 50 *ppm *CO applied during days 3-8 of pregnancy augmented the weight of fetuses (d) and placentas (e). Sacrificing the animals immediately after CO treatment (day 8 of pregnancy) revealed no differences in the levels of COHb and tHb among the groups (f and g). Data are shown as median. **p < 0.01, as analyzed by the non-parametric Mann-Whitney *U*-test between two groups. e-f data are shown as mean. ** < 0,01 as analyzed by unpaired t-test.

The model chosen to explore the effect of CO application is a relevant one for studying IUGR [17]. Additionally, it has been shown that in this particular model, the HO-1 expression is diminished in placenta from mice of the IUGR combination compared to the controls [20,21]. Application of 125 *ppm *CO during days 3-5 of pregnancy slightly diminished the rate of fetal loss as compared to air-treated controls (Figure 1a). Placentas from CO-treated animals were all normal; fetuses were not. In maternal blood, carboxyhemoglobin (COHb) or total hemoglobin (tHb) levels were not modified by this treatment (Figure 1b and 1c). Fetal death was not characterized as in the controls by resorbed, hemorrhagic feto-maternal units but by abnormalities in the fetuses (smaller size, abnormal color, disintegrated tissue) as representatively showed in Figure 1d and 1e. This suggests that albeit having a rather positive effect on placenta and partially protecting from fetal death, this particular CO dose may be not the adequate one. Next, we treated pregnant mothers with a lower CO concentration (50 ppm) during the same time period and observed no undesired effects of the gas on the surviving fetuses. CO at this time frame and concentration, however, did not diminish the rate of fetal loss (Figure 1f). Neither COHb nor tHb levels in maternal blood were modified by this treatment (Figure 1g and 1h). We next tested whether 50 *ppm *in an extended period of time, namely during implantation and during early placentation (days 3-8) may be effective in preventing fetal loss We observed that fetal loss was positively influenced by CO treatment, it decreased from 30% in air treated controls to 0% in mice receiving 50 *ppm *CO (Figure 2a), without any apparent toxic effect on the fetuses. COHb and tHb levels from maternal blood were not modified by this treatment (Figure 2b and 2c). Interestingly, the application of 50 *ppm *CO during implantation and placentation significantly augmented the weight of fetuses (Figure 2d) and placentas (Figure 2e). Normal pregnant animals (BALB/c-mated CBA/J females) which were exposed to CO did not show any differences regarding pregnancy outcome, COHb levels, placental/fetal weight or appearance of the fetuses after the treatment as compared to air-exposed animals (*data not shown*). Because the animals were sacrificed 6 days after the CO treatment and it may be possible that a putative augmentation in the COHb levels is not longer detectable at this time point, we next applied CO at the same conditions (50 *ppm *during days 3-8 of pregnancy) but sacrificed the animals immediately after treatment. The COHb and tHb levels at this time point were also comparable to the controls (Figure 2f and 2g). Thus, this is another parameter indicating that CO at this concentration is not toxic. Thus, this dose of CO does not provoke any deleterious effect in normally developing pregnancies, while having a positive effect on placental and fetal weight while it reduces significantly the intrauterine fetal death.

### Inhaled CO at 50 ppm during days 3-8 of pregnancy has anti-inflammatory effects

**Figure 3:**
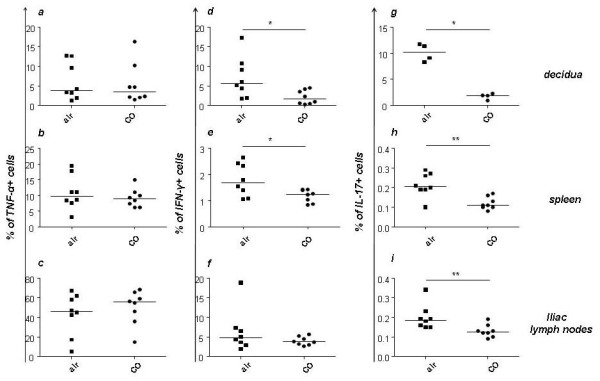
**CO application diminished the levels of IFN-γ^+^, IL-17^+ ^but not TNF-α^+ ^cells**. The percentage of TNF-α***^+ ^***cells in decidua (a), spleen (b) and iliac lymph nodes (c) of pregnant mice was not modified by treatment with 50 *ppm *CO. The percentage of IFN-γ***^+ ^***cells in decidua (d) and spleen (e) were significantly decreased after 50 *ppm *CO treatment as compared to animals which received mixed air. No differences were found for the levels of these molecules in lymph nodes (f). CO treatment lead to a significant diminution in the percentage of IL17^+ ^cells in decidua, spleen and lymph nodes (g, h, i). Pregnant animals were treated either with mixed air or 50 *ppm *CO during days (3-8 of pregnancy) and sacrificed on day 8 of pregnancy. Percentage of cells positive for TNF-α***^+^***, IFN-γ^+ ^or IL-17^+ ^were analyzed by flow cytometry and expressed as dot plots showing the median. Statistical significances were analyzed by the non-parametric Mann-Whitney *U*-test between two groups. *:p < 0.05 and **: p < 0.01.

We next asked whether the *in vivo *CO application provoked any changes in the cytokine profile secreted by lymphocytes at the periphery or at the fetal-maternal interface. It is known e.g. that Th1 and Th17 cytokines are associated with pregnancy loss (18, 22) while Th2 cytokines are known to support pregnancy (23). We could not observe any changes in the secretion of TNF-α among the groups. Immune cells from spleen and decidua secreted less IFN-γ in those pregnant mice previously exposed to CO as compared to the air-treated controls (Figure 3d and 3e) as compared to animals treated with mixed air. CO was also able to diminish the secretion of IL-17 in cells from decidua, spleen and iliac lymph nodes (Figure 3 g-i). Additionally, CO diminished the percentage of CD4^+^CD69^+ ^activated cells while increasing the frequency of CD4^+^FoxP3^+ ^regulatory T cells in iliac lymph nodes (*data not shown*) These data suggest that the positive effect of CO on placental and fetal weight as well as on fetal survival is associated with a shift of the immune response to a non-inflammatory, protective one.

### Inhaled CO diminished the apoptosis rate in placenta while augmenting the levels of the cytoprotective molecule Bag-1

**Figure 4:**
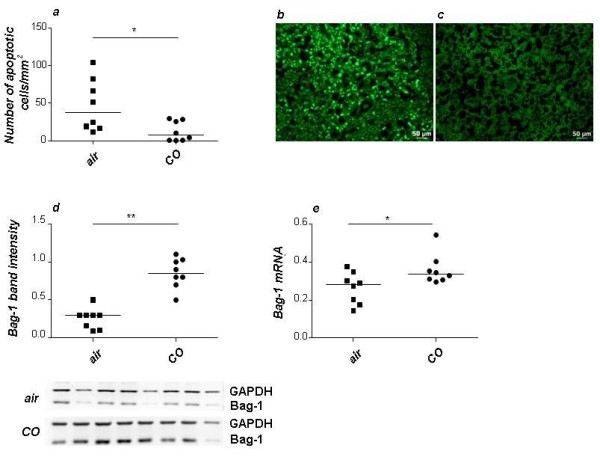
**CO treatment significantly diminished cell death in placental tissue and up-regulated the expression of the anti-apoptotic molecule Bag-1**. (a) Analysis of DNA fragmentation by TUNEL staining carried out in samples containing placental tissue from day 14 of pregnancy revealed a significant diminution in the number of apoptotic cells in the CO-treated group compared to air treated group. Representative pictures show the abundant presence of TUNEL positive cells in placental tissue from control animals (b) or the almost absent TUNEL positive cells in placental tissue from CO-treated animals (c). The bars represent 50 μm. (d-e) The expression of the anti-apoptotic gen Bag-1 was up-regulated in placental tissue from animals receiving CO treatment compared to control animals at both protein (d) and mRNA (e) level. Representative picture showing a western blot is included in (d). Data are presented as median. Statistical significances were evaluated by the non-parametric Mann-Whitney-*U *test. *:p < 0.05.

We analyzed the effect of CO on apoptosis rate as well on the expression of several molecules of the apoptosis pathway. The analysis of apoptotic cells by TUNEL carried out on samples from day 14 of pregnancy reveals that the apoptosis rate is significantly down-regulated in the CO-treated group compared with the air-treated group (Figure 4a-c). We wondered whether exposure to CO may diminish apoptosis by stimulating the expression of tissue-protective molecules, like Bag-1, Bcl-2 or Bcl-xL in placental tissue. We found the anti-apoptotic molecule Bag-1 to be significantly up-regulated after treatment with CO when compared with the air-treated group. This was true at protein as well as at mRNA level (Figure 4d-4e). The mRNA levels of the other measured antiapoptotic/cytoprotective molecules were not influenced by CO treatment (*data not shown*). Thus, CO further supports the well being of the placenta by inhibiting apoptosis and augmenting the levels of Bag-1.

### Application of CO had a pro-angiogenic effect in the placenta

**Figure 5:**
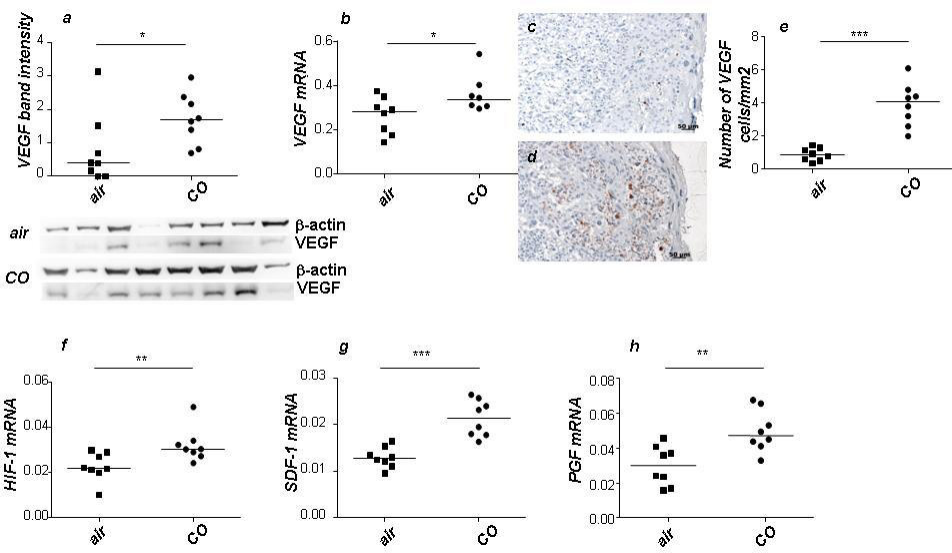
**Carbon monoxide application augments the levels of pro-angiogenic molecules in the placenta**. Inhalation of 50 *ppm *CO led to a significant augmentation in the expression of the pro-angiogenic molecule VEGF in placenta samples at protein level (a) as well as at mRNA level (b). This was also true for VEGF protein levels as confirmed by immunohistochemistry, which clearly shows an intense staining in trophoblast cells from animals treated with CO (d) which was not the case for air treated control animals (c). Quantification of the numbers of cells in each group revealed a significant up-regulation of VEGF positive cells in placentas from CO-treated mice (e). CO further induced an up-regulation in the levels of reported pro-angiogenic molecules HIF-1 (f), SDF-1 (g) and PGF (h) at mRNA level as compared to air-treated control animals. Data are presented as median. Statistical significances were evaluated by the non-parametric Mann-Whitney *U*-test between two groups. *: p < 0.05 and **: p < 0.01 and ***: p < 0.001. Bars represent 50 μm.

**Figure 6:**
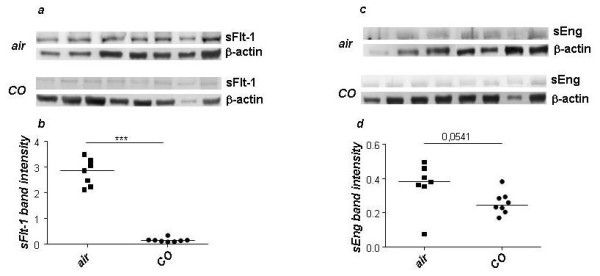
**Carbon monoxide application diminishes the levels of SFlt-1 and sEng in the placenta**. Inhalation of 50 *ppm *CO led to a significant diminution in the expression of the VEGF soluble receptor sFlt-1 at protein level (a, b). CO treatment also diminished the protein levels of sEng as analyzed by Western Blot (c, d). Data are presented as single dots and medians. Statistical significances were evaluated by the non-parametric Mann-Whitney *U*-test between two groups. ***: p < 0.001.

Because of the known effect of CO on angiogenesis, we next investigated whether the *in vivo *treatment with CO had an effect on levels of molecules positively associated with angiogenesis at trophoblast level. We concentrated on the expression of vascular endothelial growth factor (VEGF) that promotes the repair of injured vessels by stimulating angiogenesis and re-establishing vascular integrity [24]. CO inhalation significantly enhanced VEGF protein and mRNA levels (Figure 5a-5b). To identify the cells expressing VEGF, we next conducted immunohistochemistry. We observed that, in line with the Western Blot and PCR results, placentas from CO-treated mice exhibited an intense staining of VEGF in trophoblasts (Figure 5d), which was not the case for air-treated controls (Figure 5c). The quantification of the number of VEGF^+ ^trophoblasts confirmed this (Figure 5e). We next analyzed whether CO treatment modifies the levels of hypoxia-inducible factor (HIF)-1α, which is a transcription factor regulating the expression of several angiogenic factors and inducing angiogenesis [25]. HIF-1α is also a primary regulator of VEGF induction in hypoxic conditions and recent *in vitro *studies demonstrated that CO can stabilize HIF-1α [26]. In comparison to air-treated females, HIF-1α mRNA levels were upregulated in those mice who inhaled CO (Figure 5f). Recent data implied HO-1 derived CO as a critical regulator of stroma cell-derived factor (SDF)-1 mediated angiogenesis [27]. Real time RT-PCR revealed significantly elevated levels of SDF-1 mRNA in placentas from CO-treated animals (Figure 5g). We could also observe a positive effect of the *in vivo *CO treatment in the elevation of placenta growth factor (PGF, Figure 5h), which promotes angiogenesis as well. Additionally, CO treatment significantly diminished the levels of soluble VEGF receptor, the fms-like tyrosinkinase (sFlt-1) as well as of soluble endoglin (sEng) as it is shown in Figure 6a, b and 6c, d respectively. Hence, CO has a pro-angiogenic effect which may contribute to support a vital development of placenta.

### Application of CO provoked a diminution in the levels of complement deposition

**Figure 7:**
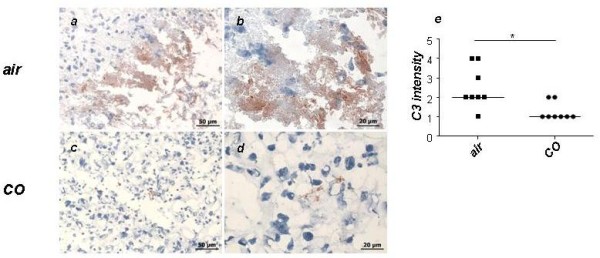
**CO treatment significantly diminished complement (C3) deposition in the placenta**. Analysis of C3 deposition after CO or air treatment showed a positive effect of CO in avoiding C3 deposition as analyzed by immunohistochemistry (a-d) and analyzed semiquantitatively (e). (a) and (c) show representative pictures from each group at 40X while (b and d) show pictures taken with a 100X objective. Data are presented as median. Statistical significances were evaluated by the non-parametric Mann-Whitney-*U *test. *:p < 0.05.

Because the adenoviral-mediated overexpression of HO-1 increased decay accelerating factor (DAF) expression prevented C3 deposition and complement-mediated lysis [28], we wondered whether the modulation of complement activation by CO may ameliorate placental vascular injury and their associated abnormal pregnancy outcomes. We tested the intensity of C3 staining in placentas from air or CO-treated mice and observed that CO breathing significantly prevented C3 deposition as observed in placentas from CO-treated mice compared to placentas from mice that were exposed to mixed air (Figure 7).

## Discussion

One of the leading causes of perinatal morbidity and mortality is intrauterine growth restriction (IUGR) [29,30]. This has further serious consequences as predisposition to lifelong increased risk of hypertension, cardiovascular disorders, renal disease among others [31,32]. Thus, there is an urgent need for solutions to avoid or reduce the occurrence of IUGR.

During human, rat and mouse pregnancies the expression of heme oxygenase-1 (HO-1) is highly induced at trophoblast level [19,20,33,34] whereas miscarriages and pre-eclampsia were showed to be associated with low HO-1 expression in the placenta [19,35,36]. IUGR is characterized as well by diminished HO-1 levels at the fetal-maternal interface [37]. We have recently reported that mice deficient in *Hmox1 *present aberrant placentation followed by a clear phenotype of IUGR and subsequent intrauterine fetal death [12]. This phenotype is caused by the accumulation of free heme and can be totally prevented by the inhalation of carbon monoxide (CO), an important metabolite of the heme breakdown by HO-1 [12]. Thus, HO-1 emerges as a key regulator of placenta physiology and fetal development, while CO overtakes the center of the scene. Having demonstrated the ability of CO to prevent IUGR and fetal death caused by insufficient HO-1 levels [12], we concentrated on analyzing the therapeutic potential of CO in a clinically relevant model of IUGR as in humans a relationship between IUGR and low HO-1 activity has been widely demonstrated [37].

Here, we show that the success and effectiveness of the therapy are highly dependent on both the doses and the time frame of CO application. While a relatively high dose (125 *ppm*) during embryo implantation can apparently prevent fetal loss, it presents undesired effects on the fetuses. A lower doses (50 *ppm*) at the same time frame (days 3-5 of pregnancy) is not toxic but is not efficient in preventing fetal death. The same low doses (50 *ppm*) applied longer, namely during implantation and placentation (days 3-8 of pregnancy) can effectively prevent fetal death while being not toxic. We recently showed that the application of 50 *ppm *during days 5-8 (placentation) also prevents fetal death [12]. It is therefore tempting to speculate that the dose and application period of CO is dependent on the concentration and/or activity of heme oxygenases in the placenta. CO application to heterozygote *Hmox1*^+/- ^mice during days 3-8 can prevent fetal death and restore the otherwise altered proportion of *Hmox1*^+/+^, *Hmox1*^+/- ^and *Hmox1*^-/- ^progeny. This clearly works via normalization of the placentation as we could observe *in vivo *[12]. In the present study, we observed a positive effect of CO on placental and fetal weight in an animal model of IUGR, which goes in line with the results obtained for HO-1-deficient animals [12]. We discard toxicity related to hypoxia after observing no changes in the levels COHb. This was also true for animals that were sacrificed immediately after treatment to analyze the immediate effect of CO application. Normal pregnant animals (Balb/c-mated CBA/J females) treated with low CO did not show any differences regarding pregnancy outcome. Normal pregnant controls did not show any abnormalities after CO treatment as well. Accordingly, rat pups whose mothers were chronically exposed to low doses of CO during pregnancy showed no brain damage [38].

Having confirmed that also in a clinically relevant model CO can positively affect pregnancy and rescue those fetuses in danger of developing IUGR and dying intrauterine depending on the dose and time frame of application, we wondered which pathways would be activated after exposure to this gasotransmitter. We found that CO breathing suppressed local inflammatory Th1 and Th17 responses which are known to be associated with pregnancy loss [18,22,23]. Thus, CO further contributes to pregnancy success by shifting the immune response to a non-inflammatory, protective one. It is known that the protective effects of HO-1 are related with anti-apoptosis [14]. We found that this may be also true for pregnancy, as HO-1-upregulation lead to augmented levels of Bag-1 and less apoptotic cells in the placenta [21,39]. Here, we confirmed that, as for accounted for HO-1 augmentation, CO application diminished the apoptosis rate in placentas and upregulated the levels of Bag-1, known to be anti-apoptotic. Treatment of placental villous explants with carbon monoxide had a positive effect which relied on anti-apoptosis [40]. CO seems therefore to be directly implied in placental physiology and thus in placentation and fetal growth by inhibiting apoptosis. We next concentrated on the effect of CO on angiogenesis, a process which is essential for placentation. Besides, CO is known to positively influence angiogenesis in several models [41,42]. It has been widely demonstrated that the induction of HO-1 activity positively contributes to the regulation of the maternal vascular tone during pregnancy [43]. CO was proposed to be involved in angiogenesis by stimulating the production of vascular endothelial growth factor (VEGF) in human microvessel and vascular endothelial cells [44-46]. Moreover it was shown that SDF-1 promotes angiogenesis *in vitro*, which is mediated by HO-1 and CO [27]. Our results revealed that *in vivo *CO treatment significantly upregulates both VEGF and SDF-1 expression in placenta. Placenta growth factor (PGF) was proposed to be involved in angiogenesis and is known to be diminished in cases of IUGR in humans [47,48]. We found here that the treatment of animals with IUGR not only rescued fetuses from death and augmented their weight but also augmented the levels of PGF in placenta. This may indicate PGF as one of the placental targets of CO, whose augmentation contribute to the well-being of the placenta and thereby to fetal survival. The idea of CO contributing positively to angiogenesis in the placenta is further supported by the data we obtained when analyzing sFlt-1 and sEng. It was already known that HO-1 and CO inhibit soluble Flt-1 [36]. Girardi and colleagues found elevated sFlt-1 levels in this particular mouse model [49], which we found to be inhibited after CO breathing. Similarly, the application of CO resulted in diminished sEng. This clearly confirms the positive role of CO in angiogenesis.

Placentation and embryonic development occurs predominantly in a hypoxic environment [50,51], being HIF-1α of crucial importance in this process [52]. HIF-1α was proposed to regulate HO-1 expression after myocardial injury [53], and CO was reported to regulate HIF-1α, suggesting a feed-back regulatory loop [26]. We were able to confirm an upregulation in the levels of HIF-1α mRNA in placenta from mice which were treated with CO. It is therefore tempting to speculate that transient diminished O_2 _levels, necessary for implantation [50] up-regulates HIF-1α locally which will in turn induce the expression of cytoprotective genes to avoid tissue damage at this particular time point and to protect the embryo against oxidative stress. Tissue cytoprotection is of particular importance in the placenta as ensures the survival of the tissue and thereby determines the adequate fetal support. The CBA/J x DBA/2J model is longer known to be dependent on complement activation [49]. We observed that CO application conferred protection against complement deposition as we analyzed by measuring C3 in the placenta. The same had been previously observed for adenoviral-mediated HO-1 overexpression [28]. Complement modulation may explain the positive effects of the gas in terms of vascular injuries as we observed that CO application normalized placenta histology and restored pregnancy [12].

In conclusion, we have confirmed the positive effects of CO on pregnancy outcome in an animal model of IUGR which is clinically relevant. We show that the effectiveness of CO-gas therapy depends on the careful selection of the dose and time frame to be applied. This is of extreme importance for the putative design of clinical trials. We show that the positive effects of CO on pregnancy, highlighted by prevention of fetal death and increased fetal weight, are associated with an anti-inflammatory local immune response, with tissue protection, anti-apoptosis and pro-angiogenesis.

## Competing interests section

The authors declare that they have no competing interests.

## Authors' contributions

TEM carried out experiments, performed statistical analysis and contributed to manuscript writing. PAC built the CO chambers, supervised CO-experiments, carried out experiments and contributed to manuscript preparation. IW, MLZ and SL contributed to experiments, HDV contributed to manuscript drafting, FJ supervised experiments, performed statistical analysis and participated in manuscript writing. ACZ conceived the study, provided the funds, participated in its design and coordination, wrote the paper. All authors read and approved the final manuscript.
